# Site-Specific Protein Modifications by an Engineered Asparaginyl Endopeptidase from *Viola canadensis*

**DOI:** 10.3389/fchem.2021.768854

**Published:** 2021-10-22

**Authors:** Yu Chen, Dingpeng Zhang, Xiaohong Zhang, Zhen Wang, Chuan-Fa Liu, James P. Tam

**Affiliations:** ^1^ School of Biological Sciences, Nanyang Technological University, Singapore, Singapore; ^2^ Synzymes and Natural Products Center, Nanyang Technological University, Singapore, Singapore; ^3^ Nanyang Institute of Structural Biology, Nanyang Technological University, Singapore, Singapore

**Keywords:** asparaginyl endopeptidases, peptide asparaginyl ligases, ligase-activity determinant, *Viola canadensis*, cell surface labeling, protein engineering

## Abstract

Asparaginyl endopeptidases (AEPs) or legumains are Asn/Asp (Asx)-specific proteases that break peptide bonds, but also function as peptide asparaginyl ligases (PALs) that make peptide bonds. This ligase activity can be used for site-specific protein modifications in biochemical and biotechnological applications. Although AEPs are common, PALs are rare. We previously proposed ligase activity determinants (LADs) of these enzymes that could determine whether they catalyze formation or breakage of peptide bonds. LADs are key residues forming the S2 and S1′ substrate-binding pockets flanking the S1 active site. Here, we build on the LAD hypothesis with the engineering of ligases from proteases by mutating the S2 and S1′ pockets of VcAEP, an AEP from *Viola canadensis*. Wild type VcAEP yields <5% cyclic product from a linear substrate at pH 6.5, whereas the single mutants VcAEP-V238A (Vc1a) and VcAEP-Y168A (Vc1b) targeting the S2 and S1′ substrate-binding pockets yielded 34 and 61% cyclic products, respectively. The double mutant VcAEP-V238A/Y168A (Vc1c) targeting both the S2 and S1′ substrate-binding pockets yielded >90% cyclic products. Vc1c had cyclization efficiency of 917,759 M^−1^s^−1^, which is one of the fastest rates for ligases yet reported. Vc1c is useful for protein engineering applications, including labeling of DARPins and cell surface MCF-7, as well as producing cyclic protein sfGFP. Together, our work validates the importance of LADs for AEP ligase activity and provides valuable tools for site-specific modification of proteins and biologics.

## Introduction

Asparaginyl endopeptidases (AEP), also known as legumain and vacuolar processing enzymes (VPE), belong to the C13 family of cysteine proteases, which break peptide bonds after Asn/Asp (Asx) residues (Abe et al., 1993; Bottari et al., 1996; Shimada et al., 2003). AEP is initially synthesized as an inactive precursor which undergoes an acidic auto-activation in the vacuole (plants) or lysosome (mammals) (Müntz and Shutov 2002; Dall and Brandstetter 2016). AEPs are particularly well studied in plants and are multifaceted enzymes that display three distinct enzymatic functions: proteolysis, splicing, and ligation.

AEPs were discovered as endopeptidases in the early 1990s, but were also shown to be splicing enzymes that post-translationally modify the circularly permutated lectin concanavalin A (Min and Jones 1994; Nonis et al., 2021). Recently, certain AEPs were shown to be potent peptide Asx-specific ligases that can serve as bioprocessing enzymes for maturation of cyclic peptides (Nguyen et al., 2014; Bernath-Levin et al., 2015; Harris et al., 2015; Yang et al., 2017; Jackson et al., 2018; Zauner et al., 2018; Harris et al., 2019; Hemu et al., 2019; Hemu et al., 2020). As such, AEPs play important roles in processing seed storage proteins to release nutrients and bioactive peptides, forming seed coats, regulating programmed cell death, and generating host-defense antimicrobial and anti-fungal peptides (Hara-Nishimura, Inoue and Nishimura 1991; Saska et al., 2007; Hatsugai et al., 2015; Dall and Brandstetter 2016; Loo et al., 2016; Yamada et al., 2020).

Of particular interest to chemical biology is the discovery that AEPs have potent and specific ligase activity for forming Asx-peptide bonds. Our laboratory termed AEP-type ligases that can reverse the enzymatic direction from proteolysis to ligation as peptide Asn/Asp-specific ligases (PALs) (Nguyen et al., 2014; Hemu et al., 2019; Hemu et al., 2020; Tam et al., 2020). Butelase-1, the prototypic PAL, was isolated from a cyclotide-producing plant *Clitoria ternatea,* a plant which is known locally as bunga telang from which the name butelase is derived (Nguyen et al., 2014). Butelase-1 is highly efficient in ligating various Asx-containing peptides and proteins at pH 4–6.5, an acidic pH range which generally favors proteolysis by legumains (Nguyen et al., 2015b). Consequently, butelase-1 has become a model for engineering an AEP to a PAL. In addition, butelase1-like PALs are valuable tools for engineering and site-specific modification of proteins, and for theranostics. Such PALs can drive macrocyclization, orthogonal ligation, antibody-drug conjugation, and protein-protein fusion reactions (Nguyen et al., 2015a; Nguyen et al., 2015b; Cao et al., 2015; Cao et al., 2016; Hemu et al., 2016; Nguyen et al., 2016; Bi et al., 2017; Wang et al., 2021; Zhang et al., 2021).

AEPs and PALs share similar structures that include a core domain, linker region, and cap domain, with identical catalytic triads composed of Cys, His and Asn (Supplementary Figure S1). To date, 16 unique AEP and PAL structures have been reported in the protein data bank. However, their superimposed structures with <1.2 Å deviations show little difference and yield no clear clues about the enzymatic directionality toward protease or ligase activity (Supplementary Figures S2, S3, Supplementary Tables S1, S2). To understand the molecular determinants underpinning the directionality of AEP and PAL activity, our laboratory has focused on the substrate-binding sites flanking the S1 pocket. We used the Schechter and Berger’s nomenclature of protease, substrate-binding pockets (Sn) which are the sub-sites of protease beside the active site (S1 pocket), to represent the region on the surface of an enzyme that can interact with peptide substrate residues (Pn) with specificity (Supplementary Figure S4) (Abramowitz, Schechter and Berger 1967). AEPs and PALs show high amino acid sequence conservation around the catalytic S1 pocket and have minor differences in the S2 and S1′ binding pockets. These sequence differences are termed ligase activity determinants (LADs) (Hemu et al., 2019). According to the LAD hypothesis, critical residues in the S2 and S1′ binding pockets that flank the catalytic S1 site are essential to steer the directionality of a legumain towards hydrolase or ligase activity (Hemu et al., 2019). Modification of these critical residues by site-directed mutagenesis was shown to either affect the enzymatic efficiency or alter the enzymatic directionality of AEPs. Our team performed extensive mutagenesis on OaAEP1b, an AEP originated from *Oldenlandia affinis* (Yang, et al., 2017). We found that the C247A mutation displayed enhanced kinetic efficiency relative to wild type. Accordingly, we coined the term “gate-keeper”, a term preceding our use of “LAD1”, to describe the importance of this site in controlling ligase activity. Recently, our group discovered asecond determinant for ligase activity which we named LAD2. We showed the importance of LAD2 for controlling ligase activity of AEPs by generating several AEP mutants derived from *Viola yedoensis* (VyPAL3-Y175G) and *Viola canadensis* (VcAEP-Y168A) (Hemu et al., 2019). Also, we successfully engineered butelase-2, which displays dominant protease activity at pH 4–6.5, to exhibit ligase activity, also at acidic pH, by mutagenesis of key LAD residues (Hemu et al., 2020). However, additional validation to strengthen the LAD hypothesis is needed.

Here we report engineering of VcAEP, an AEP from the plant *Viola canadensis*, based on the LAD hypothesis to reverse its enzymatic direction from an asparaginyl endopeptidase to a ligase. VcAEP is a dual-functional AEP that displays both protease and ligase activities. Our previous work has shown that modification of LAD2 site improved ligase activity of VcAEP. In the current study, we performed mutagenesis targeting the VcAEP S2 and S1′ pockets individually and jointly. We show that these mutants displayed enhanced ligase activity and diminished protease activity. Furthermore, we show that a double mutated VcAEP at both LAD sites is a highly efficient ligase and useful as a tool for protein labeling, sfGFP cyclization, and cell-surface labeling.

## Materials and Methods

### Data-Mining Search of AEP Analogs

The core domains of butalase-1 and OaAEP1b were used to perform a data-mining search to discover novel AEPs having ligase activity. Results of this search were further narrowed in a second search that specified Val/Ile/Cys at “gate-keeper” sequences. VcAEP has 68.2% sequence identity and 85.7% similarity with butelase-1 and 65.9% identity and 89.1% similarity with OaAEP1b. VcAEP has a Val at the “gate-keeper” position, and thus is considered to be a ligase.

### Expression, Purification, Activation of VcAEP

The full sequence of VcAEP was inserted into pET28a(+) and an N-terminal His-Ub tag was added to facilitate purification. The recombinant construct was transformed into *E. coli* SHuffle T7 cells, which were cultured at 30°C to OD_600_ 0.5. Then, 0.1 mM IPTG was added to induce VcAEP protein expression at 16°C for ∼18 h. Cell pellets were collected and lysed in lysis buffer (50 mM HEPES, 300 mM NaCl, 1 mM MgCl_2_, 1 μl/25 ml DNase I, 0.1% TritonX-100, 1mM PMSF, pH 7) with sonication. After centrifugation, the supernatant was collected and subjected to a three-step purification, including immobilized metal affinity chromatography, ion-exchange chromatography, and size exclusion chromatography. Activation of purified proenzyme was performed under acidic conditions (20 mM phosphate buffer, 0.5 mM N-Lauroylsarcosine, pH 4.5) at 37°C for 15–30 min. In addition, the activation was conducted by adding 1% or 5% acetic acid to adjust the pH to 4–4.5 with 0.5 mM N-Lauroylsarcosine, at 4°C for 10–16 h. The active form of the enzyme was purified by size exclusion chromatography using acidic buffer (20 mM HEPES, 150 mM NaCl, 1 mM EDTA, 1 mM DTT, 5% glycerol, pH 4). The purified active form of the enzyme was stored at −80°C. In-gel digestion followed by MS/MS *de novo* sequencing was performed to characterize autocleavage sites in the C terminus.

### pH-Dependent Cyclization

To determine the optimal pH for cyclization activity of VcAEP and mutants, reaction mixtures containing the active enzyme (200 nM for wild type and 100 nM for Vc1a-1c) and substrate (4 μM for wild type, 20 μM for Vc1a, 50 μM for Vc1b, and 100 μM for Vc1c) were incubated at 37°C for 15 min according to the catalytic efficiency. The reaction buffers contain 20 mM phosphate, 1 mM EDTA, 1 mM DTT. The wild-type VcAEP displays slow enzymatic activity compared to the mutants, and no product was observed in 15 min using the same enzyme:substrate ratio as the mutants. Thus, for the wild type VcAEP, we increased the enzyme concentration from 100 to 200 nM. Similarly, we decreased the substrate concentration to 4 μM. Reaction products were analyzed by calculating the peak areas of MALDI-TOF mass spectrometry.

### Kinetics Study Using FRET-Mediated Assay

A kinetics study of enzyme cyclization and ligation was conducted using fluorescence resonance energy transfer (FRET) at 37°C and pH 6.5. Fluorescence was recorded with a BioTek Cytation 5 cell imaging multimode plate reader at excitation and emission wavelengths of 390 and 460 nm, respectively. The initial rates V_0_ (μM/s) for different substrate concentrations were calculated using the Michaelis-Menten equation. Vmax and Km and consequently kcat and kcat/Km were calculated using GraphPad Prism. All FRET substrates were synthesized by standard Fmoc-based solid-phase peptide synthesis on rink amide resin (Alsina et al., 2000). EDANS was introduced by using Fmoc-Glu(EDANS)-OH. DABCYL was directly coupled to ε-amine of Lys residue in dry DCM containing 2x DIEA of DABSYL for 2 h, at room temperature. All peptides were cleaved in 95% TFA/2.5% H_2_O/2.5% TIS cleavage solution and purified by preparative RP-HPLC.

### Applications

The synthetic fluorescent peptide EDANS-ANGI contains a signal “NGI” that can be recognized by ligases. DARPin9_26 was recombinantly expressed with an N-terminal His tag and TEV cleavage site. TEV cleavage exposes an N-terminal dipeptide “GL” that can function as an incoming nucleophilic attack group. The reaction was conducted at 37°C and pH 6.5 with 20 nM Vc1c and 4 μM DARPin9_26. DARPins that are successfully ligated with fluorescent peptide produce a green color under UV radiation.

SfGFP is used as an example of Vc1c-mediated protein circularization. Acyclic sfGFP was recombinantly expressed with additional PAL-recognizing tags (N-terminal Met-Ile and C-terminal Asn-Ser-Leu-His_6_). The reaction was conducted at 37°C, pH 6.5, in the presence of 25 μM GFP and 0.5 μM Vc1c. The products were determined by high-resolution ESI-MS.

Application of Vc1c for MCF-7 cell surface labeling was performed as follows. First, MCF-7 cells cultured in plates were detached and resuspended in phosphate buffer (PBS, pH 7.4). Then, 50 μM of fluorescein-peptide and 200 nM of ligase were added to the solution and incubated at 37°C for 1 h. The cells were then washed 5 times with PBS to remove residual peptides and were resuspended in PBS buffer. FITC channels were used to detect fluorescein peptide-labeled cells.

## Results and Discussion

### Expression, Purification, and Activation of VcAEP

**FIGURE 1:**
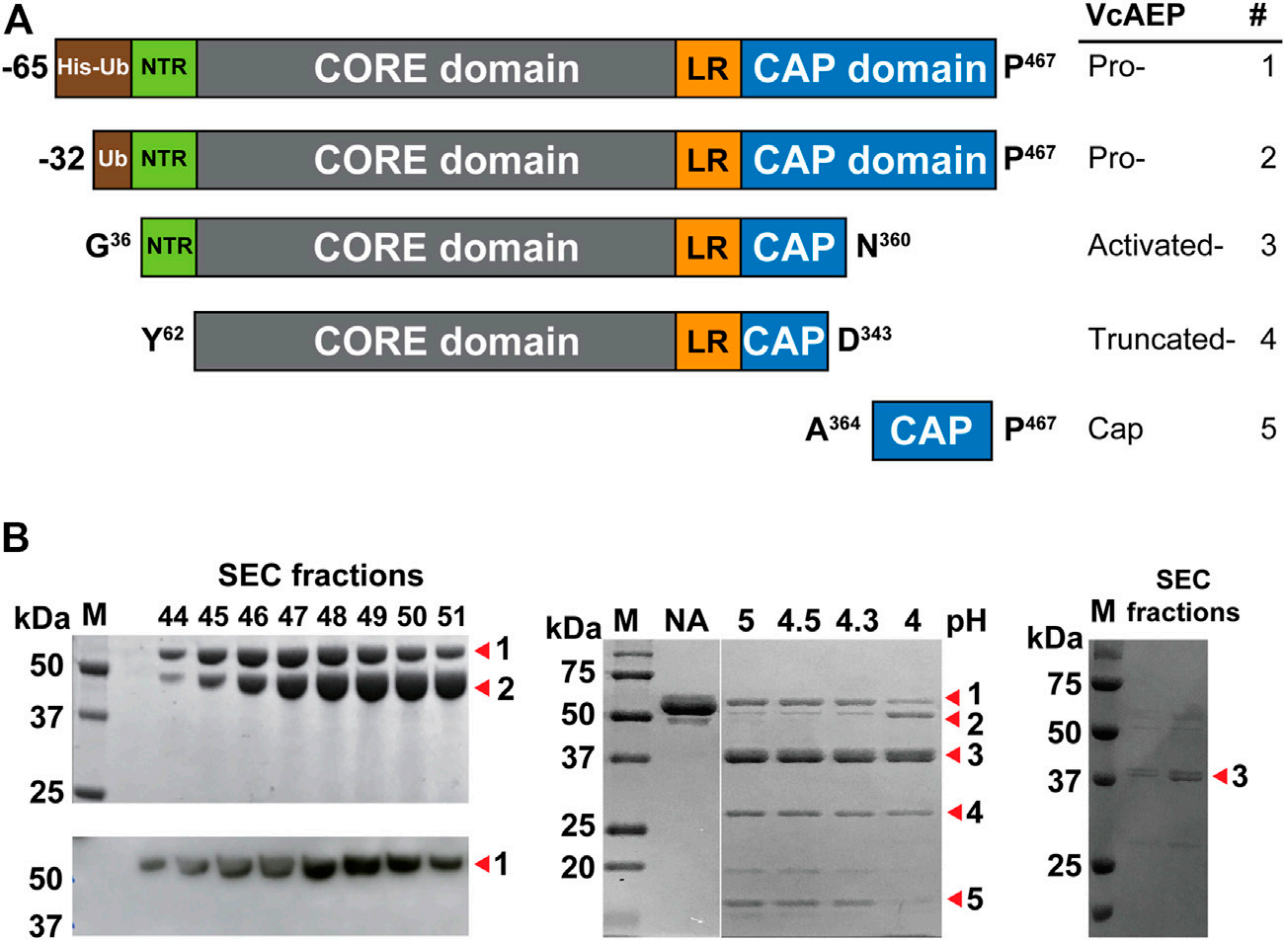
Purification and activation of VcAEP. **(A)** Schematic representation of recombinant VcAEP constructs. Pro-VcAEP 1: full-length proenzyme form expressed with His-Ub-VcAEP (His = His6 tag, Ub = ubiquitin) and ending at P467. Pro-VcAEP 2: full-length VcAEP without the His-tag. Activated-VcAEP 3: active form of VcAEP after acidic autoactivation that begins with G36 and ends with N360. Truncated-VcAEP 4: truncated form of VcAEP, starting with Y62 and ending with D343. Cap 5: cleaved cap domain after acidic autoactivation, starting with A364 and ending with P467. The processing sites were determined by in-gel trypsin digestion followed by MS/MS *de novo* sequencing. NTR, N-terminal region; LR, linker region. **(B)** Purification, expression and activation of VcAEP. Left panel: analysis of purified fractions obtained from SEC chromatography, SDS-PAGE (upper) and western blot (lower). SDS-PAGE shows two bands, Pro-VcAEP 1 and Pro-VcAEP 2 (see panel A), with the lower band 2 being the VcAEP proenzyme without the His tag. Middle panel: SDS-PAGE analysis of the lauroylsarcosine-mediated acidic autoactivation of Pre-VcAEP 1 and Pro-VcAEP 2 and its products Activated-VcAEP 3, Truncated-VcAEP 4, and Cap 5, performed at pH 4–5 at 37°C for 30 min to generate Activated-VcAEP 3 (∼37 kDa). NA: no activation. Right panel: SDS-PAGE analysis of purified Activated-VcAEP 3 by SEC chromatography using pH 4 buffer.

The VcAEP sequence was obtained by data mining using the core domains of butelase-1 and OaAEP1b. VcAEP has 68.2% sequence identity and 85.7% similarity with butelase-1 and 65.9% identity and 89.1% similarity with OaAEP1b. To purify recombinant VcAEP, the cDNA sequence containing a His-Ub (hexa-histidine-ubiquitin) tag was synthesized and cloned into pET28a(+) (Figure 1A). The plasmid was expressed in SHuffle T7, an *E. coli* strain with enhanced folding capacity. The affinity-purified proenzyme of VcAEP was obtained using immobilized metal affinity chromatography (IMAC) followed by ion exchange (IEX) and size exclusion chromatography (SEC).

Similar to the previously reported OaAEP1b (Yang et al., 2017), the VcAEP proenzyme was obtained in two forms with or without a His-Ub tag (proform **1** and **2**) (Figures 1A,B left, and Supplementary Figure S5). Both forms were around 50 kDa on SDS-PAGE (Figure 1B, left upper), but only the proform **1** with a His-Ub tag was detected on western blot using an anti-His antibody (Figure 1B, left lower). The active form of VcAEP (activated form, **3**) was generated by incubation at 37°C for 30 min with 0.5 mM lauroylsarcosine to promote acidic activation at pH 4.5 (20 mM sodium phosphate buffer, 1 mM EDTA), followed by purification by SEC using pH 4 buffer (Figure 1B, middle and right). Autoactivation sites were determined by LC-MS/MS sequencing of the tryptic-digested active forms. The Asn/Asp cleavage sites at both ends of the core domain were N35 in the N-terminal region (NTR) and D343/D349/N360 in the cap domain (Figure 1A and Supplementary Figure S6). The cleavage sites of the truncated form and cap domain were also determined (Supplementary Figures S7, S8). The result implies that a multi-step cleavage is involved in the VcAEP acidic auto-activation. In addition, the processed activated form **3** of VcAEP retains a short cap domain, which might be helpful for the stabilization of activated form.

### Characterization of VcAEP Activity

**FIGURE 2:**
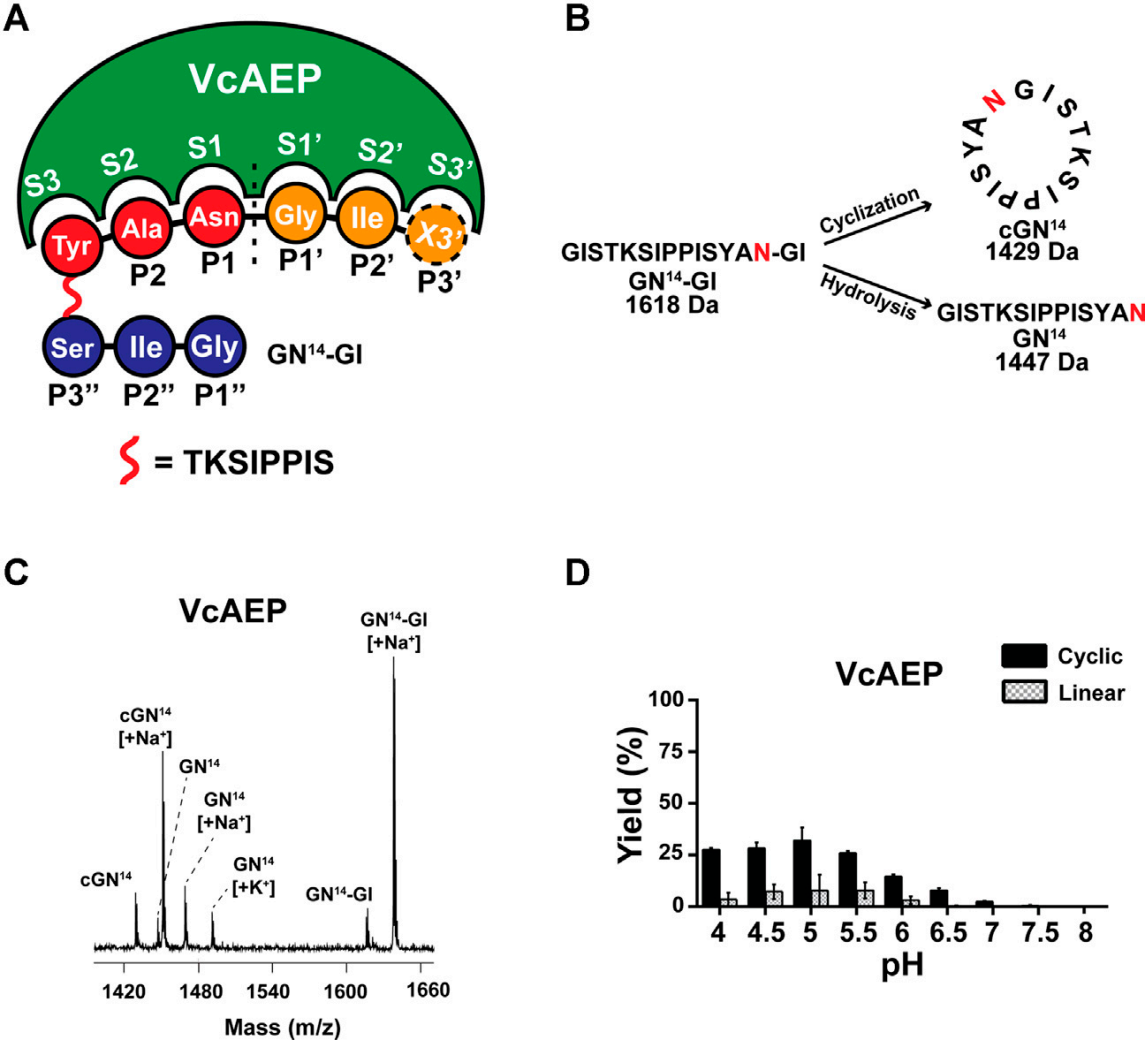
Analysis of VcAEP AEP activity. **(A)** Nomenclature of AEP/PAL and substrate, using the peptide substrate GN^14^-GI (GISTKSIPPISYAN-GI) as a model. Based on the nomenclature of substrate binding of protease proposed by Schechter and Berger, the cleavage site of the substrate is named P1 (red), and the corresponding binding pocket of the enzyme is S1. After cleavage, the amino acid residues of leaving group are P1′, P2′, P3′ Etc (orange). For PALs to cyclize or ligate substrate, the amino acid residues of the incoming group are P1″, P2″, P3″ etc., (blue). X stands for the 20 amino acids. **(B)** Schematic representations of AEP-mediated cyclization and hydrolysis of the 16-residue peptide substrate GN^14^N-GI (MW 1618 Da). The P1-Asn position is colored red. The enzymatic reaction of VcAEP yields two products: the 14-residue cyclic product cGN^14^ (1429 Da) and 14-residue linear product GN^14^ (1447 Da) with release of a GI dipeptide. **(C)** Representative MALDI-TOF mass spectrometry of the cyclic product cGN^14^ and the linear product GN^14^ generated by VcAEP together with GN^14^N-GI as the starting material. The reaction was conducted at pH 6, at 37°C for 15 min with a 1:20 molar ratio of enzyme:substrate. **(D)** Product yields of VcAEP wild type were quantified by calculating the peak areas of MALDI-TOF mass spectrometry. Gray and black bars indicate the percentage yield of linear product and cyclic product, respectively. The reactions were conducted at 37°C for 15 min with a molar enzyme:substrate ratio of 1:20 (VcAEP). Average yield and SDs were calculated from experiments performed in triplicate.

AEPs require a tripeptide recognition motif Asx-Xaa-Yaa (P1-P1′-P2′, using Schechter and Berger nomenclature) to make peptide bonds (Abramowitz et al., 1967). Generally, at the P1′ position, the substrate requirements are not stringent, although small amino acid residues are typically present. At P2′, bulky and aromatic amino acid residues are required for AEPs to carry out ligations (Figure 2A) (Nguyen et al., 2014; Jackson et al., 2020). We synthesized a 16-residue model peptide substrates GN^14^-GI (GISTKSIPPISYAN^14^-GI) to characterize the activity of VcAEP. The functional activity of VcAEP at pH 6 was initially assayed by MALDI-TOF mass spectrometry using GN^14^-GI (MW 1618 Da) to determine its ligation efficiency to yield the 14-residue cyclic product cGN^14^ (MW 1429 Da) or its proteolytic activity to yield the linear product GN^14^ (MW 1447 Da) (Figure 2B). Under given conditions, both cyclic (15%) and linear products (5%) were generated, indicating that VcAEP is a dual-function enzyme that has both protease and ligase activities (Figure 2C).

The ligase activity of AEPs and PALs is highly dependent on pH (Hemu et al., 2019; Hemu et al., 2020). Thus, we performed the pH-dependent cyclization of VcAEP over the range of pH 4–8 using GN^14^-GI. The reactions were conducted at 37°C for 15 min at the indicated pH. The yield of cyclic product (MW 1430 Da) and linear product (MW 1448 Da) were quantified in triplicate by calculating the peak areas of MALDI-TOF mass spectrometry. VcAEP displays a low ligase activity between pH 4–7 toward the peptide substrate GN^14^-GI with a high enzyme concentration of 1:20 molar ratio of enzyme to substrate (Figure 2D).

### Mutants Targeting S2 and S1′ Substrate-Binding Pockets Display Potent Ligase Activities

**FIGURE 3:**
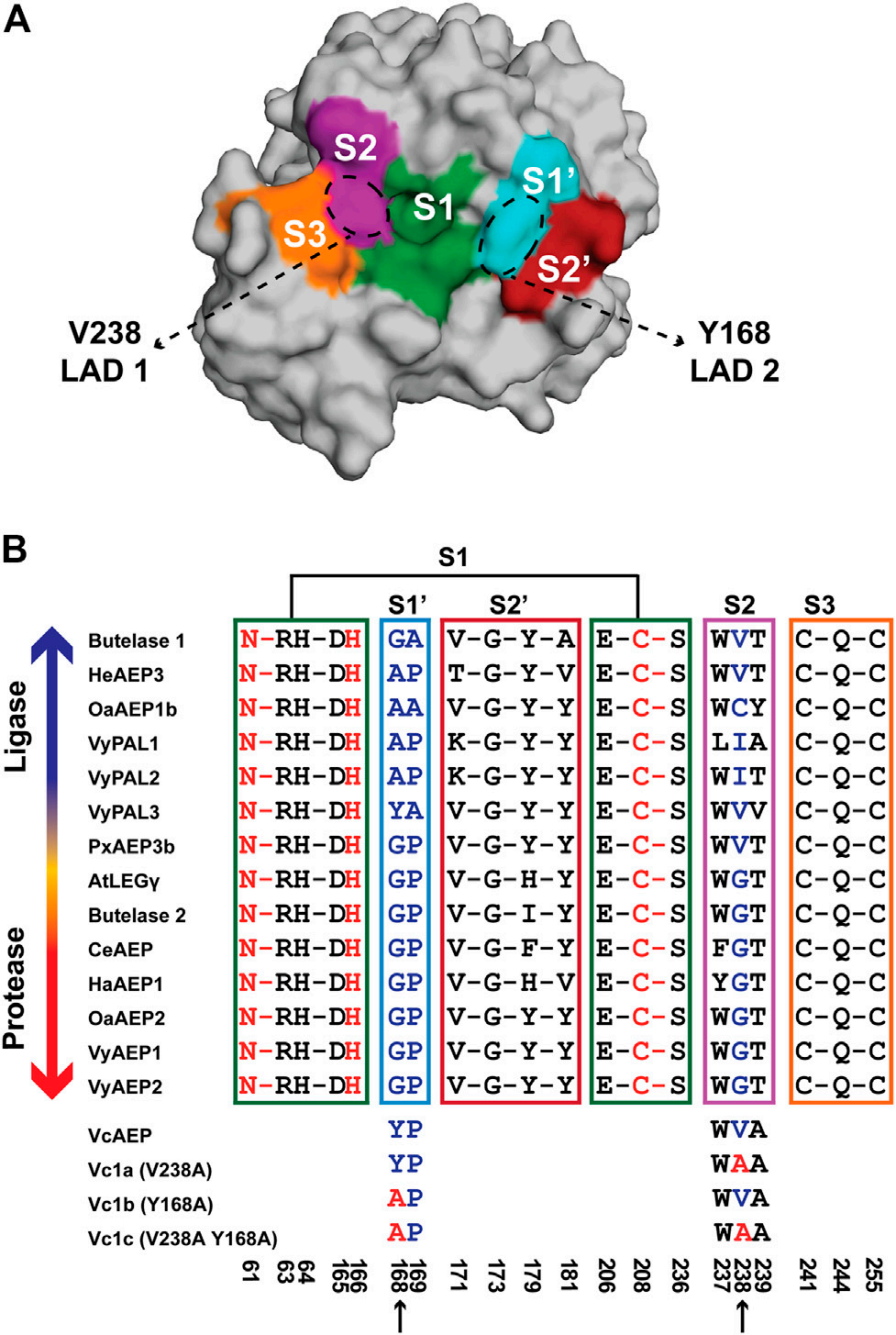
Engineering a ligase from a dual-functional AEP, VcAEP. **(A)** Proposed substrate binding pockets (S3, S2, S1, S1′, S2′) based on the crystal structure of the VcAEP proform (pdb: 5ZBI). The S3 pocket (orange) comprises residues C241, Q244, and C255, S2 (purple) W237, V238, and A239, S1 (green) N61, R63, H64, D165, H166, E206, C208, and S236, S1′ (cyan) Y168 and P169, S2′ (red) V171, G173, Y179, and Y181. **(B)** Sequence alignment of substrate-binding pockets of known AEPs for which protease activity dominates (e.g., CeAEP, butelase-2), and PALs having dominant ligase activity (e.g., butelase-1, VyPAL2). The blue arrow indicates increasing ligase activity and the red arrow indicates increasing protease activity. The three engineered VcAEP mutants having mutations at S2 (V238 using VcAEP numbering) and S1′ (Y168) sites are Vc1a (V238A), Vc1b (Y168A), and Vc1c (V238A Y168A). Color codes are the same as for **(A)** and **(B)** with S3 in orange, S2 in purple, S1 in green, S1′ in cyan, and S2 in red. The catalytic triad N61, H166, and C208 in the oxyanion hole corresponding to S1 is colored red. Key residues in the S1′ and S2 pockets for steering AEP or PAL activities (panel B) are respectively colored cyan for Y168P169, and purple for V238. Residues comprising the catalytic S1, S1′, S2′, S2 and S3 pockets are framed by a green, cyan, red, purple and orange box, respectively. Three VcAEP mutants with increasing ligase activity were engineered that target S2 and S1′ pockets (indicated by black arrows).

Sequence alignment of known AEPs and ligases shows that the S1 and S3 pockets are highly conserved. Meanwhile, the S2′ pocket contains four residues that have diverse sequences and are located relatively far from the catalytic S1 pocket (Figures 3A,B). Sequence variations of S2 and S1′ substrate-binding pockets are vital factors to control the ligase activity of AEPs. The S2 pocket, also known as the “gate-keeper” or LAD1, comprises three residues, of which the first residue is an aromatic Trp or Tyr and the third residue is hydrophobic residues Ala/Val or neutral residues Thr/Tyr. The ligases tend to have Val/Ile/Cys as the middle residue, whereas proteases often have Gly. The S1′ pocket, also known as LAD2, has two residues. Ligases often have Gly-Ala or Ala-Ala or Ala-Pro, and AEPs typically have Gly-Pro. It should be noted that the ligase activity of AEP is not solely determined by substrate-binding pockets, but rather results from cooperation of different factors (Haywood et al., 2018; Jackson et al., 2018).

Previously, our laboratory engineered eight PAL-like mutants of butelase-2 which is an AEP with protease activity at acidic pH. These PAL-like mutants targets both the LAD1 and LAD2 sites (see Figure 3B). They include Bu2a (V/GP), Bu2b (I/GP), and Bu2c (A/GP), targeting the middle residue of LAD1 motif Gly252 by substituting it with Val, Ile, or Ala, respectively. We also prepared LAD2 mutants targeting Gly182-Pro183 by substituting them with Ala, giving Bu2d (G/GA) (butelase-1-like) and Bu2e (G/AP) (VyPAL2-like). In addition, we engineered double-site-LAD mutants: Bu2f (V/AP) (VyPAL2-like), Bu2g (V/GA which is butelase-1-like), and Bu2h (V/AA whichOaAEP1b-like). The best engineered butelase-2 mutant acting as a butelase-1 was Bu2g which contains mutations in both LAD1 and LAD2 sites (Hemu et al., 2020).

To confirm our mutation study of converting butelase-2 from a protease to a ligase, we herein focus on engineering the S2 and S1′ pockets of VcAEP corresponding to the LAD1 and LAD2 sites, respectively, to promote its ligase activity and demonstrate the essential roles these factors play in AEP ligase activity. According to the LAD hypothesis, VcAEP is a bifunctional AEP with a Trp-Val-Ala sequence in the S2 pocket and thus is considered a ligase. In contrast, the S1′ pocket sequence of VcAEP is Tyr-Pro, which does not fit the criteria for ligases. To validate the LAD hypothesis, we engineered three mutants: VcAEP-V238A or Vc1a that has an Ala substituted for Val in the middle residue in the LAD1 motif at position 238 (V238A); VcAEP-Y168A or Vc1b that has the LAD2 residue Y168 changed to Ala; and VcAEP-V238A/Y168A or Vc1c, a double mutant targeting both LAD1 and LAD2 pockets (Figure 3B). We used a similar protocol to express, purify, and activate these mutants as was used for wild type VcAEP (Supplementary Figure S9). Although Vc1a and Vc1b differ by a single amino acid, they migrated differently in SDS-PAGE as bands MW <37 and >37 kDa, respectively. We do not have an explanation for this result. However, we found that Vc1c and Vc1a which differ also by a single amino acid migrated similarly in the SDS-PAGE.

To compare the ligase activity of the mutants Vc1a-1c to the wild type VcAEP, we performed the pH-dependent cyclization reactions mediated by Vc1a-1c. The ligation reactions were performed over the pH range of pH 4 to pH 8 using the peptide substrate GN^14^-GI, at 37°C for 15 min. The yield of cyclic product (MW 1430 Da) and linear product (MW 1448 Da) were quantified in triplicate by MALDI-TOF mass spectrometry.

**FIGURE 4:**
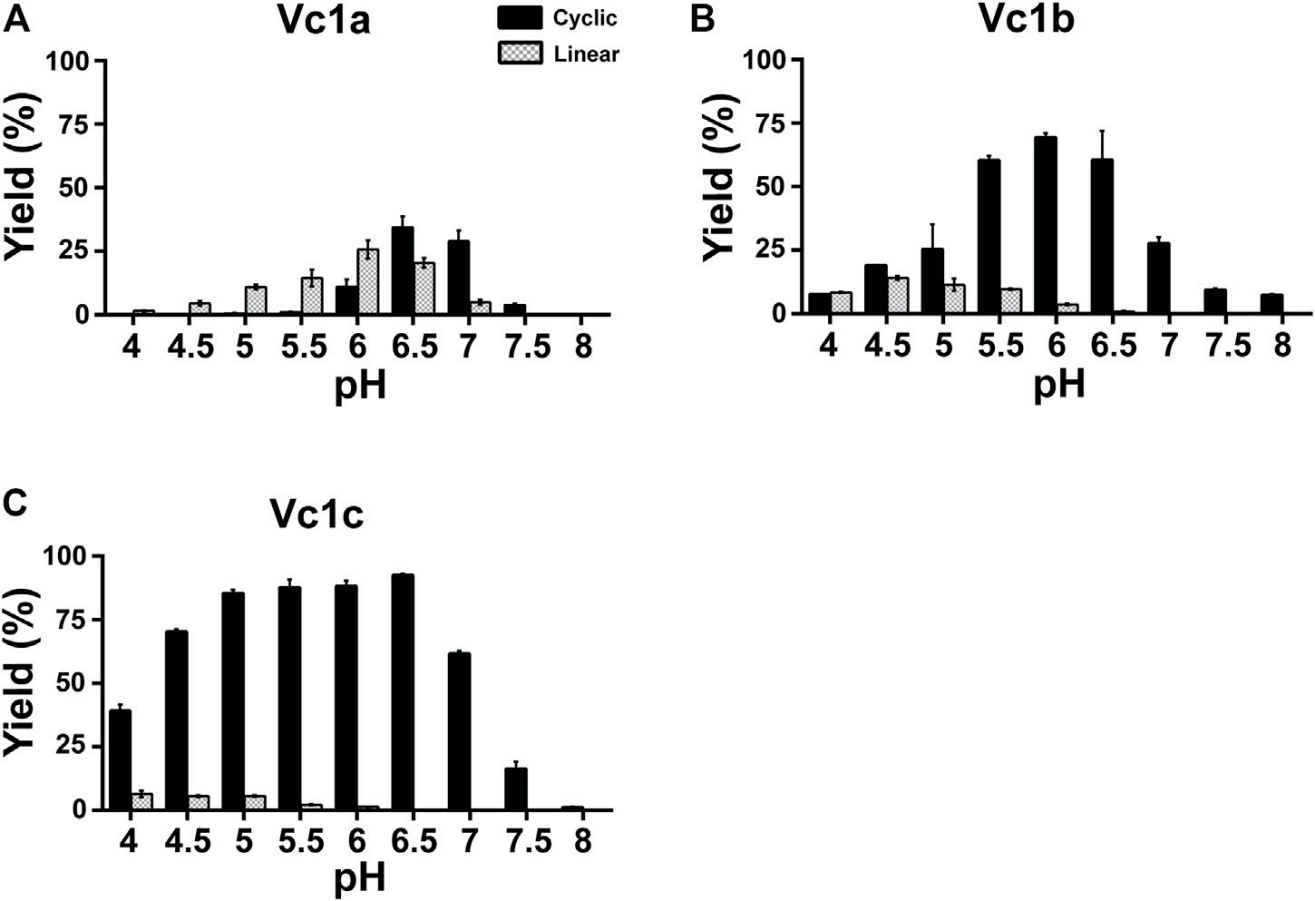
pH-dependent cyclization efficiency of VcAEP and mutants. **(A–C)** Quantitative summary of VcAEP mutant Vc1a-Vc1c product yield analyzed using MALDI-TOF mass spectrometry. Gray and black bars indicate the percentage yield of linear product and cyclic product, respectively. The reactions were conducted at 37°C for 15 min with a molar enzyme:substrate ratio of 1:200 (Vc1a), 1:500 (Vc1b), and 1:1000 (Vc1c). Average yield and SDs were calculated from experiments performed in triplicate.

Vc1a (VcAEP-V238A), a LAD1 mutant that has a V238A mutation in the S2 binding pocket, displayed dominant protease activity at pH < 6, but dominant ligase activity at pH > 6.5 using the GN^14^-GI peptide substrate at a 1:200 molar ratio of enzyme to substrate. Using the same reaction time and temperature, Vc1a consumed substrate at a much faster rate than wild type VcAEP. As such, we used a 1:200 enzyme:substrate molar ratio for Vc1a and 1:20 for VcAEP because Vc1a is a “faster” enzyme compared to VcAEP (Figure 4A).

The process for AEP and PAL to produce linear or cyclic products mainly involves two steps: formation of an S-acyl intermediate that then undergoes nucleophilic attack by a water molecule (hydrolysis) or a free amine of the peptide substrate (ligation). LAD1 is proposed to affect the formation of the S-acyl intermediate, which is the rate-limiting step, by shifting the molecular surface toward the incoming peptide substrate to affect the overall reaction rate (Hemu et al., 2020). Therefore, substitution of Ala for Val at position 238 of LAD1 is likely to accelerate the formation of the S-acyl intermediate and the overall catalysis process, including the production of linear and cyclic products.

Vc1b (VcAEP-Y168A) has a Tyr to Ala mutation in the LAD2 pocket (Y168A) and exhibited dominant ligase activity at pH > 4.5 and no visible hydrolytic product at pH > 6.5 using the peptide substrate GN^14^-GI at a 1:500 molar ratio of enzyme to substrate. At pH 6.5, about 60% cyclization yield was observed (Figure 4B). This result supports the LAD hypothesis that LAD2 plays a more direct role in determining AEP enzyme directionality. The wild type VcAEP has a Tyr-Pro dipeptide in LAD2, and the bulky Tyr would likely accelerate the departure of the leaving group, such that water molecules would have increased opportunity to attack the S-acyl intermediate and in turn generate more hydrolysis product. However, Vc1b with an Ala-Pro dipeptide in LAD2 would retain the leaving group for a longer time to allow selectively an incoming amine group to be ligated.

The double mutant Vc1c (VcAEP-V238A/Y168A) with mutations in both LAD1 and LAD2 showed the fastest activity of all four enzymes tested. Moreover, Vc1c generated >80% cyclic product between pH 5 and 6.5 using the peptide substrate GN^14^-GI at a 1:1000 molar ratio of enzyme:substrate (Figure 4C). Engineering both LAD1 and LAD2 in Vc1c integrated the advantages of substitutions at both sites to enhance catalytic efficiency of peptide bond formation. Together, our results show that both LAD mutants, Vc1b and Vc1c, display significant ligase activity at physiological pH and could be valuable for peptide/protein engineering applications.

### Kinetic Studies of VcAEP and Vc1a-1c

To figure out the catalytic potency of the VcAEP and its mutants, we determined their catalytic efficiency using the fluorescence resonance energy transfer (FRET)-base assay. FRET substrates were synthesized by standard Fmoc-based solid-phase peptide synthesis on rink amide resin (Alsina et al., 2000). The fluorophore EDANS was introduced by using Fmoc-Glu(EDANS)-OH. DABCYL was directly coupled to the ε-amine of Lys residue.

We initially performed the kinetics study for the wild type VcAEP and its mutant Vc1a using a 17-residue peptide substrate GISTKSIPPIE(EDANS)YRN-SLK(DABCYL). The determined kcat/Km of VcAEP and Vc1a using substrate were 4,464 M^−1^ s^−1^ and 14,706 M^−1^ s^−1^, respectively (Table 1). The kcat of Vc1a (0.2025 s^−1^) was about 11-fold higher than that for VcAEP (0.01863 s^−1^), indicating that Vc1a can consume substrate at a much faster rate than VcAEP. Vc1a also shows a 3-fold increase in kcat relative to VcAEP, suggesting that altering the S2 pocket influences substrate binding such that Vc1a needs more substrate than VcAEP to perform its activity.

**TABLE 1:** Kinetic parameters of VcAEP and Vc1a-1c.

	Enzyme	kcat (s^−1^)	Km (μM)	kcat/Km (s^−1^ M^−1^)	Substrate sequence	Enzyme concentration (μM)
Cyclization	VcAEP	0.01863	4.173	4,464	GISTKSIPPIE(EDANS)YRN-SLK(DABCYL)	0.438
	Vc1a	0.2025	13.77	14,700	GISTKSIPPIE(EDANS)YRN-SLK(DABCYL)	1.39
	Vc1b	0.03471	1.719	200,192	GISKPE(EDANS)SYAN-GIK(DABCYL)	0.06
	Vc1c	2.31	2.517	917,759	GISKPE(EDANS)SYAN-GIK(DABCYL)	0.044
Ligation	Vc1c	0.3761	33.68	11,166	N-terminal: RE(EDANS)AN-GI	0.044
					C-terminal: GVK(DABCYL)	

The peptide substrate GISTKSIPPIE(EDANS)YRN-SLK(DABCYL) was difficult to purify. Consequently, we synthesized a 13-residue peptide substrate GISKPE(EDANS)SYAN-GIK(DABCYL) to study the kinetics of Vc1b and Vc1c. It should be pointed out that both substrates give highly favored cyclic peptides of 14 and 10 membered rings. Compared with the turnover rate (kcat) in intra-molecular ligation for wild type VcAEP (0.01863 s^−1^), the kcat for Vc1b and Vc1c was found to increase by 2- (0.03471 s^−1^) and 120-fold (2.31 s^−1^), respectively. Together with the affinity constant Km, the catalytic efficiency (kcat/Km) of Vc1b and Vc1c in intra-molecular ligation was calculated to be 20,192 M^−1^ s^−1^ and 917,759 M^−1^ s^−1^, respectively (Table 1). In this regard, Vc1c shows a comparable cyclization activity to that of butelase-1 (GISTKSIPPYRN-SLAN) (Hemu et al., 2019). As such, Vc1c is one of the most efficient peptide ligases reported thus far.

Site-specific modifications of proteins are generally inter-molecular ligation reactions. To determine the capability of Vc1c in inter-molecular reactions, we synthesized an N-terminal peptide REAN-GI with the fluorophore (EDANS) group and a C-terminal peptide GVKR with the quencher (DABCYL) group. Calculation of Michaelis-Menten kinetics for Vc1c ligation based on FRET assay results showed a kcat/Km of 11,166 M^−1^ s^−1^ (Table 1) suggesting that the intermolecular ligation is many fold slower than intramolecular ligation which has substantially higher effective molarity than intermolecular reactions. .

### Application of Vc1c in Site-Specific Protein Modifications

The catalytic parameters showed that Vc1c displayed a substantially improved catalytic efficiency compared to wild type VcAEP. Next, we showed the versatility of the engineered Vc1c as a useful tool for chemical biology, site-specific protein modification, and cell-surface labeling.

#### Vc1c-Mediated N-Terminal Labeling of DARPin

**FIGURE 5:**
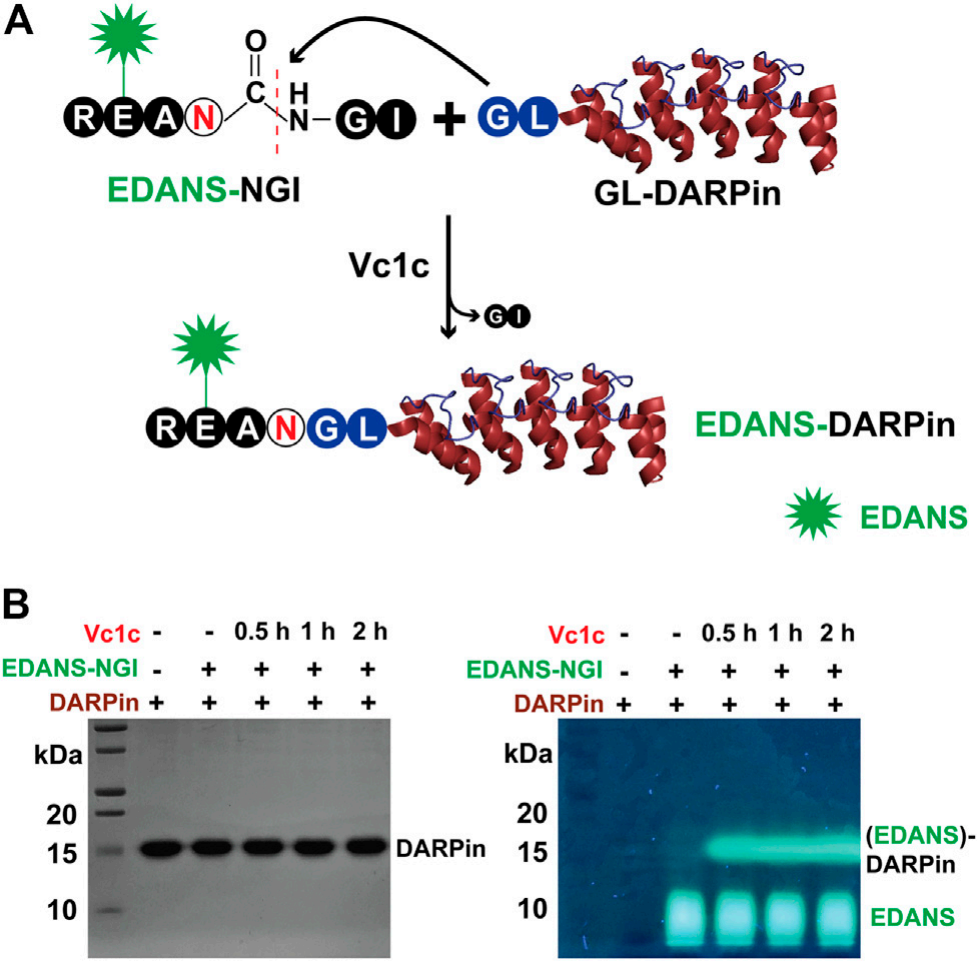
Vc1c-mediated protein labelling. **(A)** Schematic of Vc1c-mediated ligation of DARPin (generated from PDB code 2QYJ) with fluorescent peptide RE(EDANS)AN-GI. A fluorescent peptide RE(EDANS)AN-GI was synthesized and DARPin9_26 was co-expressed with an N-terminal Gly-Leu dipeptide. The ligase can recognize and cleave the C-terminal Gly-Ile of the fluorescent peptide, and ligate it to the GL-DARPin9_26 N terminus. **(B)** Left panel: SDS-PAGE gel after Coomassie blue staining showing bands for DARPin9_26 around 15 kDa; Right: SDS-PAGE gel under UV illumination. The reaction was performed at 37°C, pH 6.5 with 20 nM Vc1c and 4 μM DARPin9_26.

We first tested the activity of Vc1c in the labeling of DARPins, a genetically engineering antibody-mimetic protein based on ankyrin repeat proteins (Bork 1993). DARPins conjugated with different molecules have been used for various applications (Stumpp, Binz and Amstutz 2008). DARPin can be selected from a library to bind to any desired target, such as human epidermal growth factor receptor 2 (HER2), a transmembrane protein overexpressed on the surface of some breast cancer cells (Ross 2009). DARPins represent a new generation of protein therapeutics, and the development of various methods for DARPin labeling is of great importance. Vc1c was able to mediate DARPin9_26 N-terminal labeling. First, a fluorescence peptide RE(EDANS)AN-GI was synthesized as an N-terminal peptide. Then, GL-DARPin9_26 having the N-terminal residues “Gly-Leu” was recombinantly expressed (Figure 5A). The reaction was performed at 37°C, pH 6.5 with 20 nM Vc1c and 4 μM DARPin9_26 and was complete within 30 min. This result showed that Vc1c efficiently ligated DARPin9_26 to fluorescent peptides (Figure 5B).

#### Vc1c-Mediated GFP Cyclization

**FIGURE 6:**
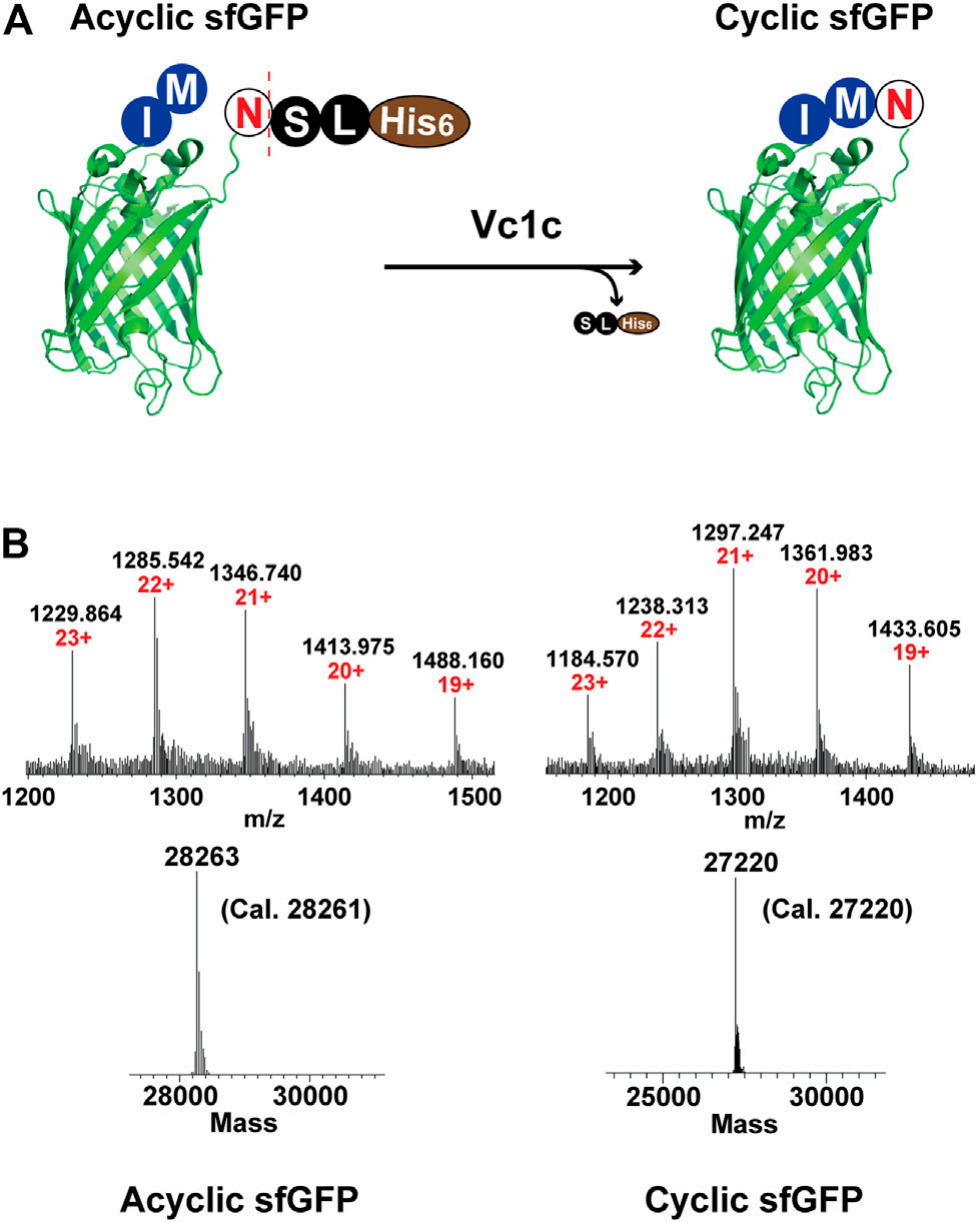
Application of Vc1c in end-to-end cyclization of sfGFP. **(A)** Scheme of ligase-mediated sfGFP (generated from PDB code 2B3P) cyclization. sfGFP was co-expressed with an N-terminal Met-Ile and a C-terminal Asn-Ser-Leu-His6. The ligase can recognize and cleave the C-terminal motif Ser-Leu-His6 and perform end-to-end cyclization of sfGFP. **(B)** Upper: ESI-MS characterization of CM-sfGFP-NSL-His6 starting material or acyclic sfGFP (left) and cyclic sfGFP (right); Lower: Deconvolution of sfGFP cyclization (CM-sfGFP-NSL-His6: Cal. 28261, Obs. 28263; Cyclic sfGFP: Cal. 27220, Obs. 27220). The cyclization reaction was performed at pH 7.0, 37°C for 30 min using 25 µM CM-sfGFP-NSL-His6 and 500 nM Vc1c.

Green Fluorescent Protein (GFP) is a widely used reporter for many applications including studies of protein folding, gene translation, and protein-protein interactions (Kalir et al., 2001; Waldo 2003; Magliery et al., 2005). A GFP variant, “super folder” GFP (sfGFP), was developed to reduce aggregation and increase folding efficiency (Pédelacq et al., 2006). In addition, the circular topology of proteins can contribute to improved stability. To demonstrate the feasibility of Vc1c to catalyze end-to-end protein cyclization, we expressed sfGFP with an additional N-terminal Met-Ile and a C-terminal Asn-Ser-Leu-His6 (Figure 6A). The cyclization reaction was performed in the presence of 25 μM GFP and 0.5 μM Vc1c. The reaction was complete within 30 min, as determined by high-resolution ESI-MS (Figure 6B).

#### Vc1c-Mediated MCF-7 Cell Surface Labeling

**FIGURE 7:**
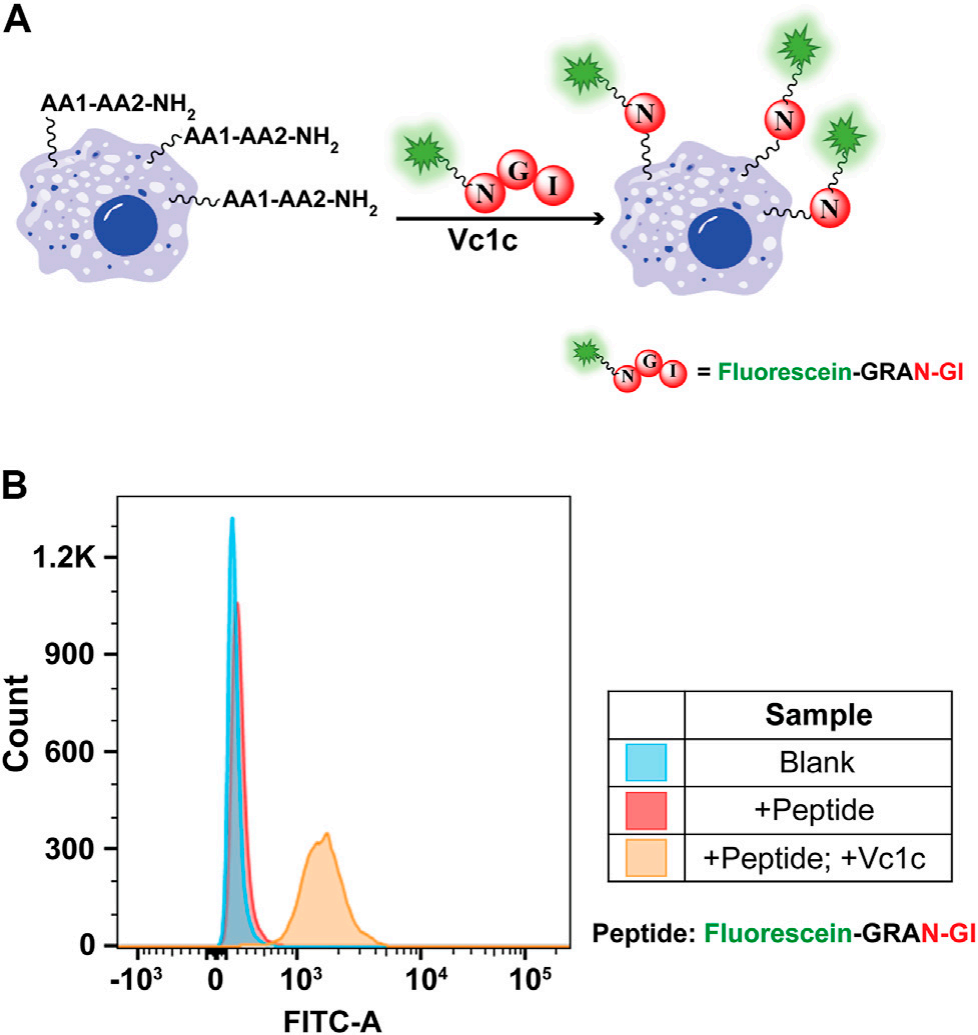
Vc1c-mediated MCF-7 cell labelling. **(A)** Schematic of MCF-7 cell surface amine labelling with the peptide fluorescein-GRANGI using Vc1c. **(B)** Flow cytometry analysis of MCF-7 labelling with the peptide fluorescein-GRANGI. Untreated cells with neither peptide nor ligase added and cells with only peptide added were used as negative controls. The reaction was conducted at 37°C for 1 h using 50 μM fluorescein-peptide and 200 nM Vc1c.

AEP-mediated cell surface labeling is a promising method for cell surface modifications (Bi et al., 2017; Harmand et al., 2021). Previous study has shown that AEP can be used for cell surface modification by specifically labeling the C-terminal hTFR-NHV (Bi et al., 2020). However, the labeling efficiency is limited due to the unsatisfactory amount of receptor protein was expressed on the cell surface. To establish a convenient and highly efficient labeling strategy, we explore the use of PAL enzyme for unspecific cell surface N-terminal amine labeling. To perform the reaction, MCF-7 cells (a typical cancer cell line) were detached and suspended in PBS buffer. The reaction was conducted using 50 μM of fluorescein-GRAN-GI and 200 nM of Vc1c at 37°C for 1 h. The cells were then washed and suspended in PBS buffer for flow cytometry with FITC channels used to detect fluorescein peptide-labeled cells (Figure 7A). MCF-7 cells were successfully labeled with Vc1c in the presence of fluorescein peptides (Figure 7B). These findings show that Vc1c is an efficient ligase for the modification of cell surface proteins, and thus this engineered protein could be valuable for biotechnology applications.

## Conclusion

In this report, we successfully engineer an AEP from *Viola canadensis* to an AEP-type ligase or PAL by mutating the residues that reside in the substrate-binding pockets flanking the S1 active site. Ligases are useful for site-specific modification proteins and cell-surface labeling which are valuable tools for biotechnology, drug development, and theranostics. A prerequisite requirement is that they can be used under mild conditions, preferably under physiological conditions. PALs fulfill this requirement. Although PALs and AEPs share similar structure and substrate specificity, PALs are rare compared to AEPs. Thus, the ability to engineer a more commonly found AEP to a PAL will provide an additional ligase with a different ligase profile to expand the repertoire of PALs which can be used alone or combined with another ligase for tandem, orthogonal, and one-pot ligation (Harmand et al., 2018; Wang et al., 2021; Zhang et al., 2021). In addition, we have found that these AEP-type ligases have different tripeptide recognition signal, substrate specificity and optimal pH for ligation reactions. Importantly, the mutant Vc1c can be expressed and purified in yields ranging from 5 to 10 mg/L of bacteria culture. The engineered ligase Vc1c is one of the most efficient ligases known to date. In addition, we show that the engineered enzyme Vc1c is a versatile tool in both peptide and protein cyclization, protein and cell-surface labeling, comparable to the natural or recombinant expressed butelase-1 (Nguyen et al., 2014; Hemu et al. 2021). Together, we provide a method to engineer a ligase and valuable tools for site-specific modification of proteins which can be further used in theranostic applications.

## Data Availability

The datasets presented in this study can be found in online repositories. The names of the repository/repositories and accession number(s) can be found below: https://www.rcsb.org/structure/5ZBI, https://www.rcsb.org/structure/2QYJ, https://www.rcsb.org/structure/2B3P, https://www.rcsb.org/structure/4FGU, https://www.rcsb.org/structure/4NOK, https://www.rcsb.org/structure/4D3Y, https://www.rcsb.org/structure/5H0I, https://www.rcsb.org/structure/5NIJ, https://www.rcsb.org/structure/6DHI, https://www.rcsb.org/structure/6IDV, https://www.rcsb.org/structure/6L4V, https://www.rcsb.org/structure/6L4W, https://www.rcsb.org/structure/6L4X, https://www.rcsb.org/structure/4D3X, https://www.rcsb.org/structure/5LUA, https://www.rcsb.org/structure/6AZT, https://www.rcsb.org/structure/5OBT.
