# Supplementary material for: Site-Specific Protein Modifications by an Engineered Asparaginyl Endopeptidase from Viola canadensis
Source: Front Chem. 2021 Oct 22;9:768854. doi: 10.3389/fchem.2021.768854 (PMC8568951; doi:10.3389/fchem.2021.768854)
Supplement: Supplementary file 1 [file DataSheet1.pdf]

(A)

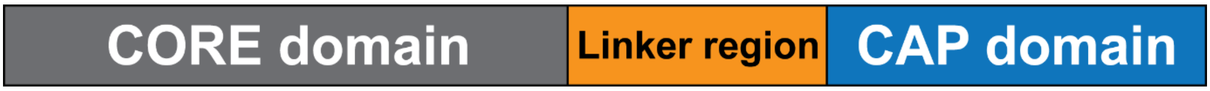

(B)

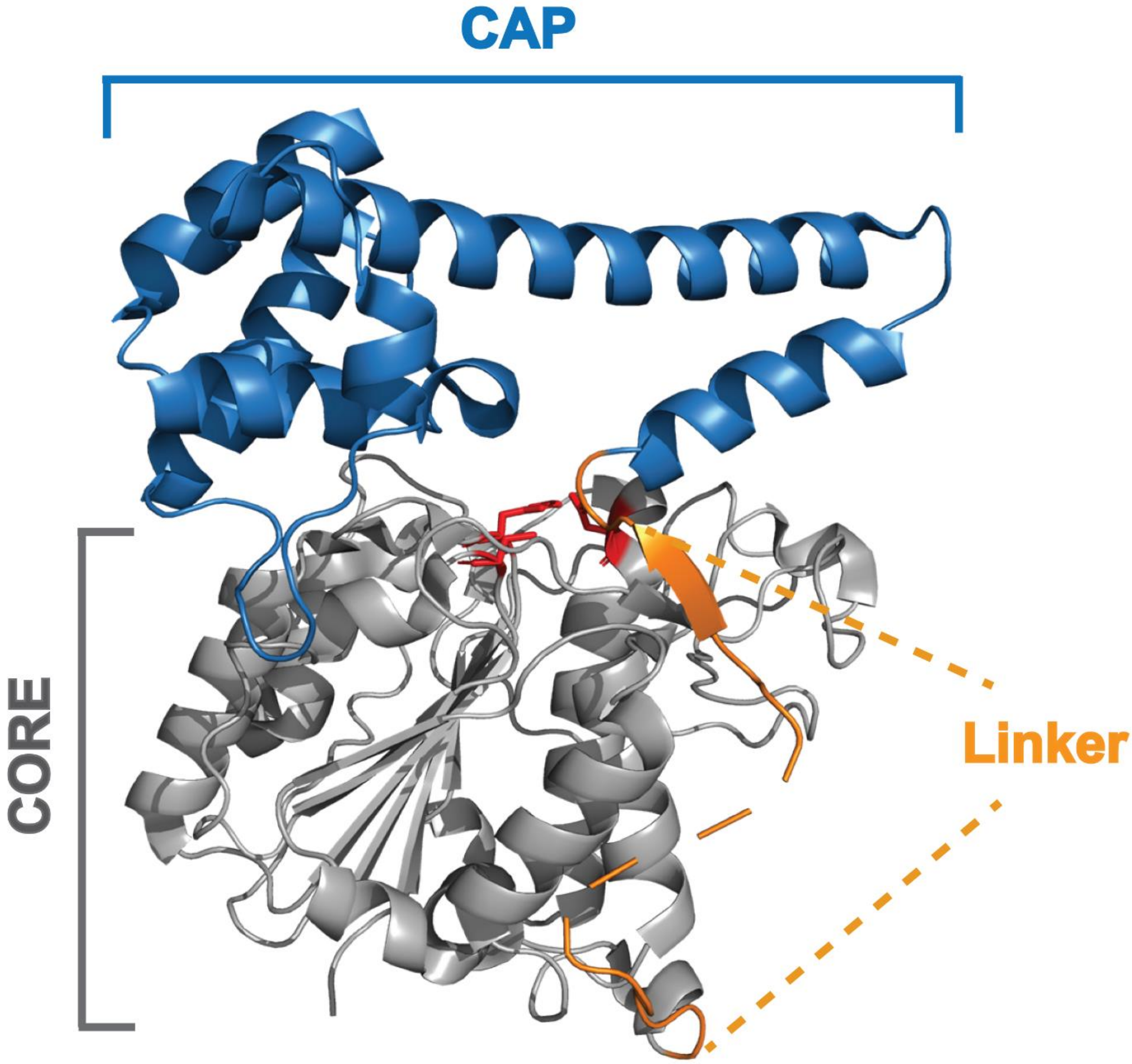

Figure S1. Structure of AEP.

(A) Diagram of AEP domains. AEP generally consist of three domains: core domain (grey), flexible linker region (orange), and cap domain (blue). **(B)** AEP structure represented by VcAEP (PDB: 5ZBI). The color codes are similar to (A). The catalytic triad in active pocket comprises Asn, His, and Cys (red).

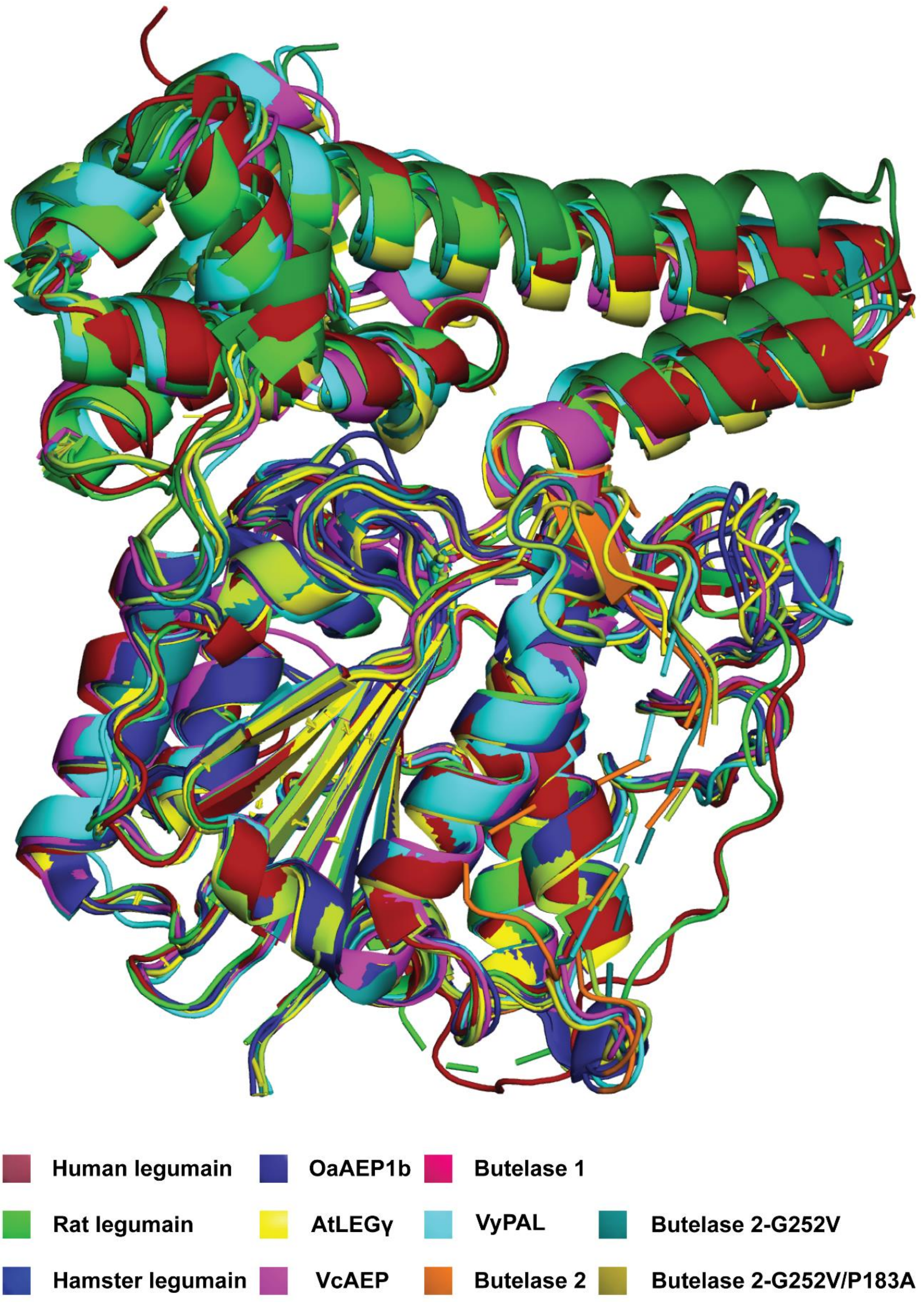

Figure S2. Structure's superimposition of 11 AEPs and PALs proenzymes.
Each color represents one single AEP structure. The color codes are shown below. The PDB codes of the included AEPs are listed below. Human legumain: 4FGU, rat legumain: 4NOK, hamster legumain: 4D3Y, OaAEP1b: 5H0I, AtLEGγ: 5NIJ, VcAEP: 5ZBI, butelase 1: 6DHI, VyPAL2: 6IDV, butelase 2: 6L4V, butelase 2-G252V: 6L4W, butelase 2-G252V/P183A: 6L4X.

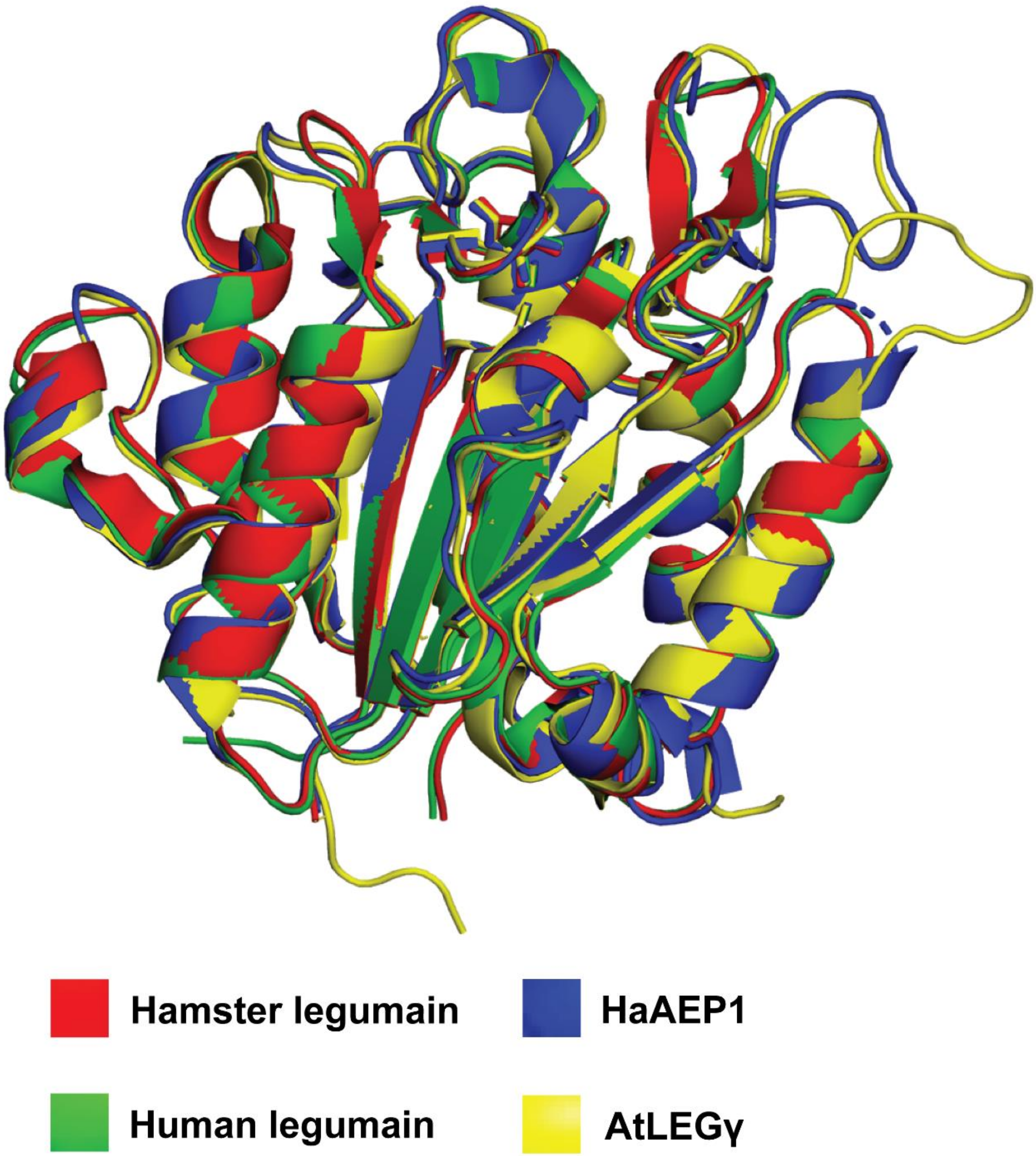

Figure S3. Structure's superimposition of 4 AEPs and PALs active forms. Each color represents one single AEP structure. The color codes are shown below. The PDB codes of the included AEPs are listed below. Hamster legumain: 4D3X, human legumain: 5LUA, HaAEP: 6AZT, AtLEGγ: 5OBT.

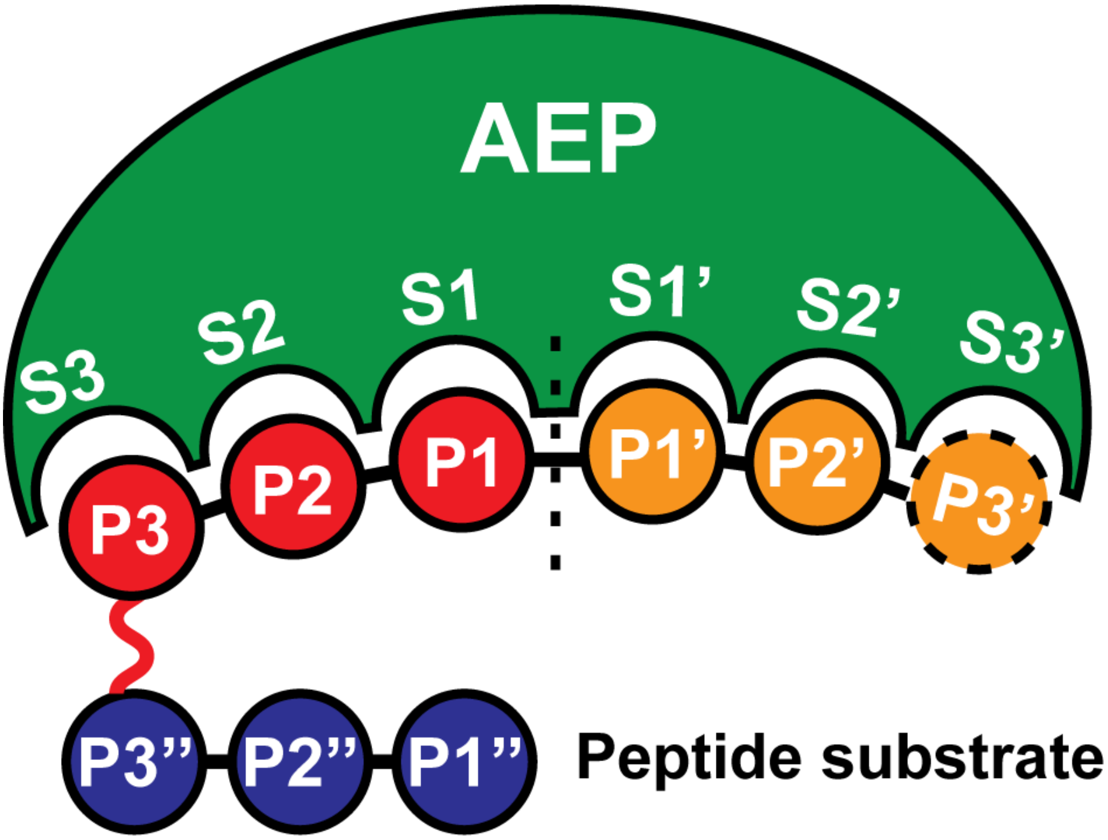

Figure S4. Nomenclature of AEP/PAL and peptide substrate. Based on the nomenclature of substrate binding of protease proposed by Schechter and Berger, the cleavage site of the substrate is named P1 (red), and the corresponding binding pocket of the enzyme is S1. After cleavage, the amino acid residues of leaving group are P1′, P2′, P3′ Etc (orange). For PALs to cyclize or ligate substrate, the amino acid residues of the incoming group are P1″, P2″, P3″ Etc (blue).

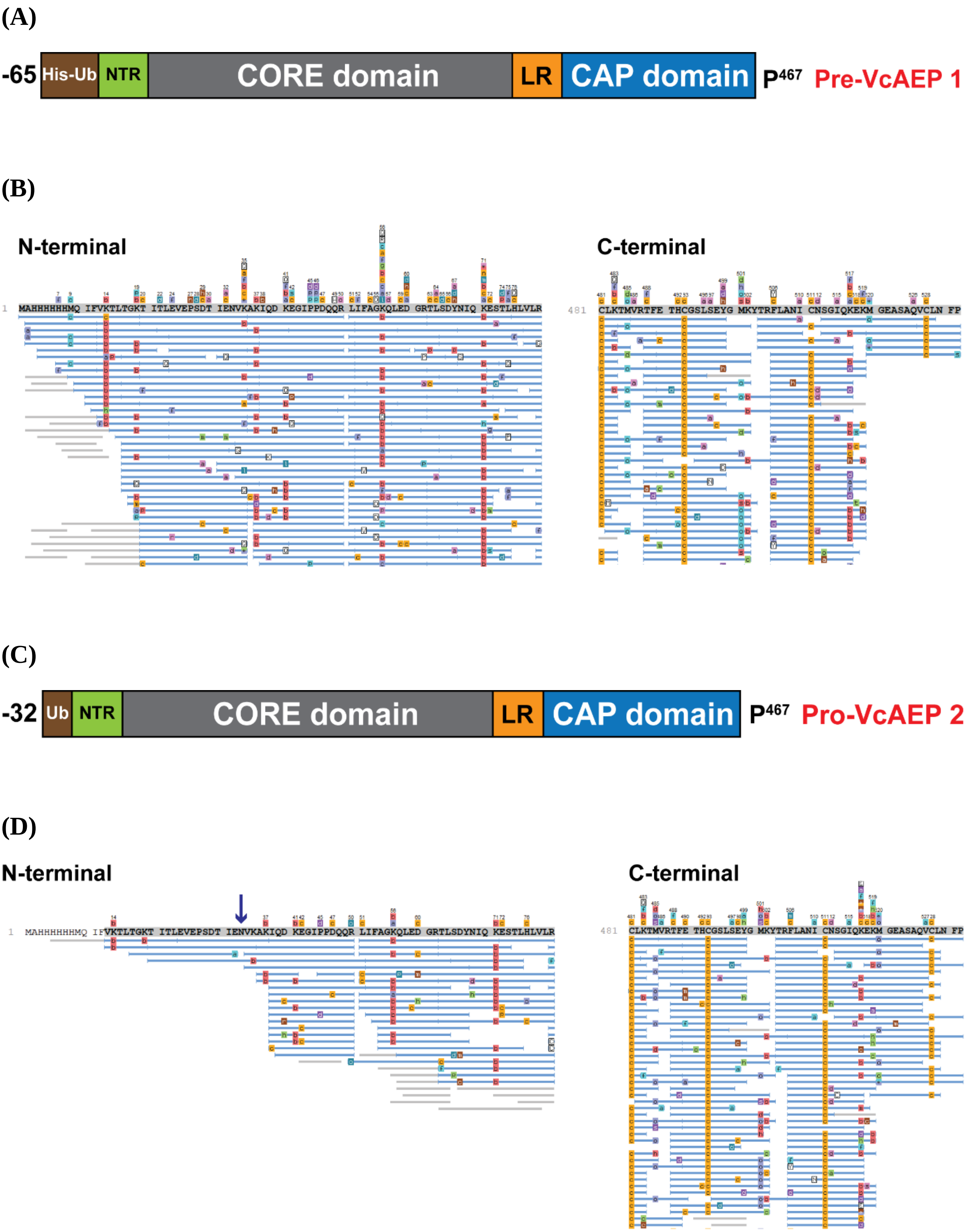

Figure S5. MS/MS *de novo* sequencing of VcAEP proenzyme.
(A) Schematic representation of Pre-VcAEP 1, the full-length proenzyme form of His-Ub-VcAEP. **(B)** The MS/MS *de novo* sequencing result of Pre-VcAEP 1 showing N- and C-terminal sequence. **(C)** Schematic representation of Pro-VcAEP 2, the full-length proenzyme form of Ub-VcAEP, lost His tag. **(D)** The MS/MS *de novo* sequencing result of Pro-VcAEP 2 showing N- and C-terminal sequence. The Blue arrow indicates the predicted processing site in N-terminal.

(A)

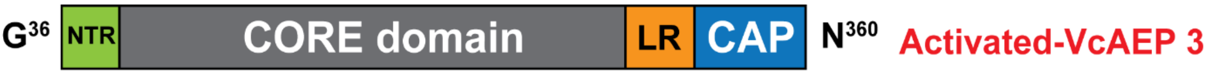

(B)

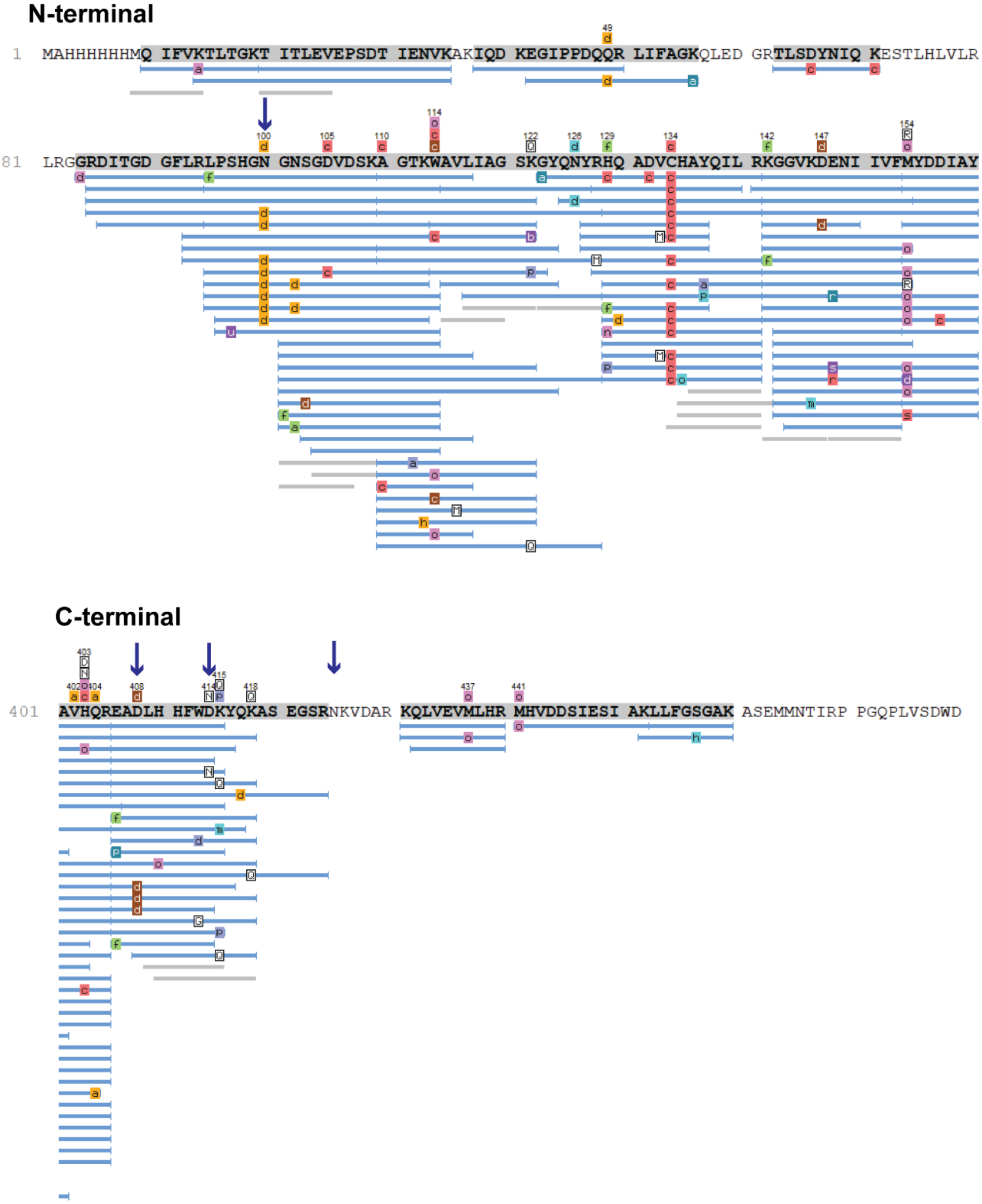

Figure S6. MS/MS *de novo* sequencing of VcAEP activated form.

(A) Schematic representation of activated-VcAEP 3, the activated form of VcAEP. **(B)** The MS/MS *de novo* sequencing result of activated-VcAEP 3 showing N- and C-terminal sequence. Blue arrows indicate the predicted processing site.

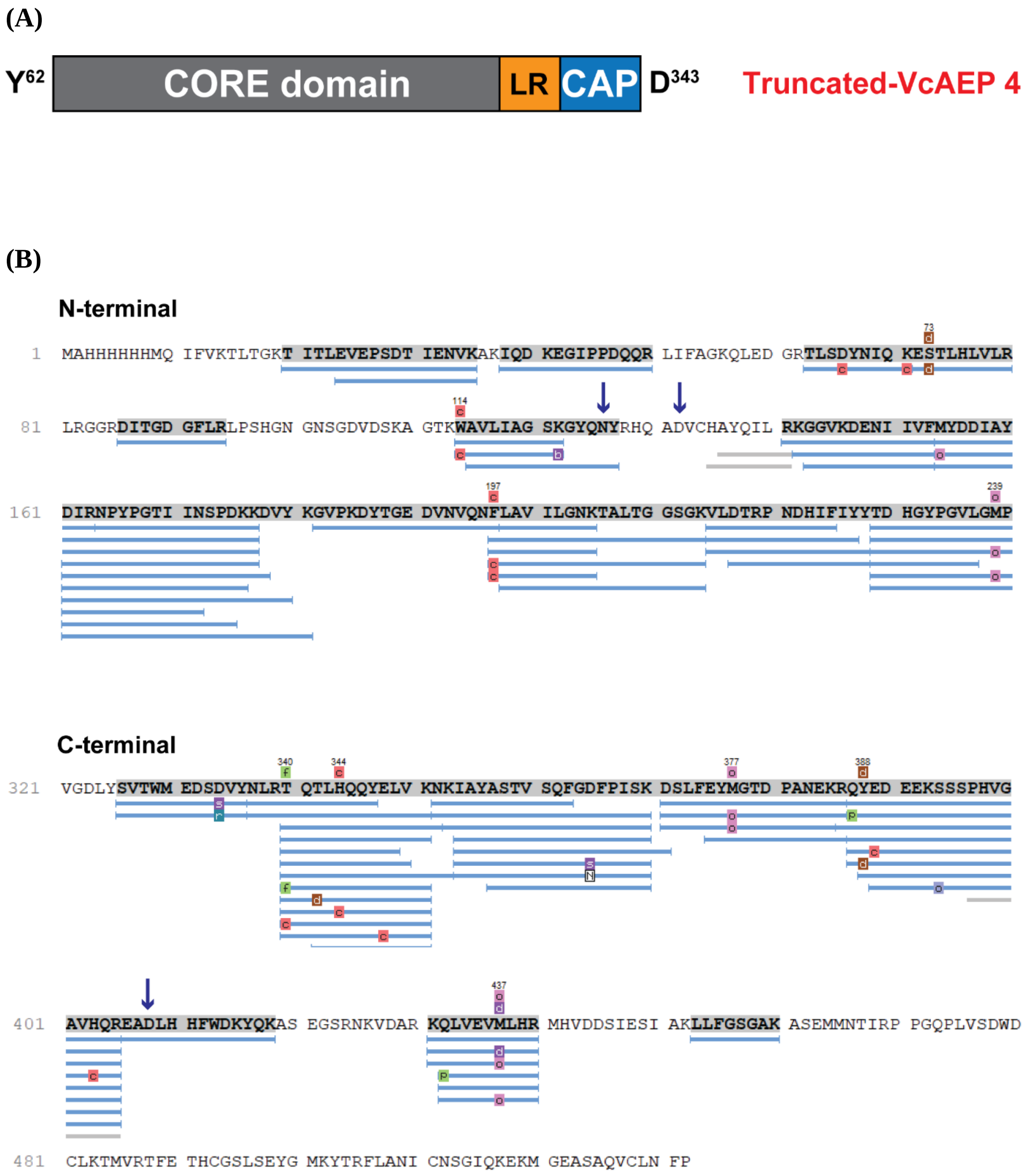

Figure S7. MS/MS *de novo* sequencing of VcAEP truncated form.

(A) Schematic representation of truncated-VcAEP 4, the truncated form of VcAEP. **(B)** The MS/MS *de novo* sequencing result of truncated-VcAEP 3 showing N- and C-terminal sequence. Blue arrows indicate the predicted processing site.

(A)

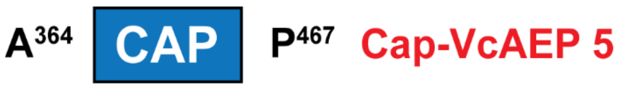

(B)

C-terminal

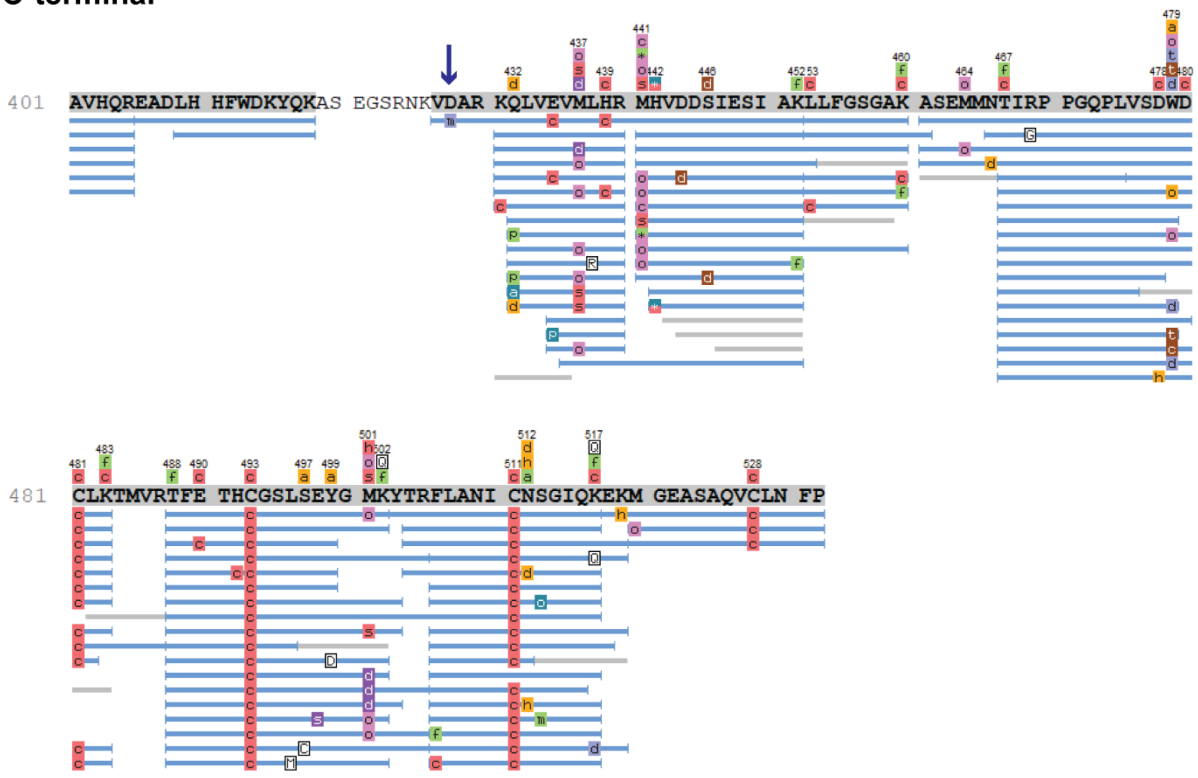

Figure S8. MS/MS de novo sequencing of VcAEP cap domain processed during VcAEP activation.

(A) Schematic representation of VcAEP cap 5. **(B)** The MS/MS *de novo* sequencing result of VcAEP cap 5 showing C-terminal sequence. The Blue arrow indicates the predicted processing site.

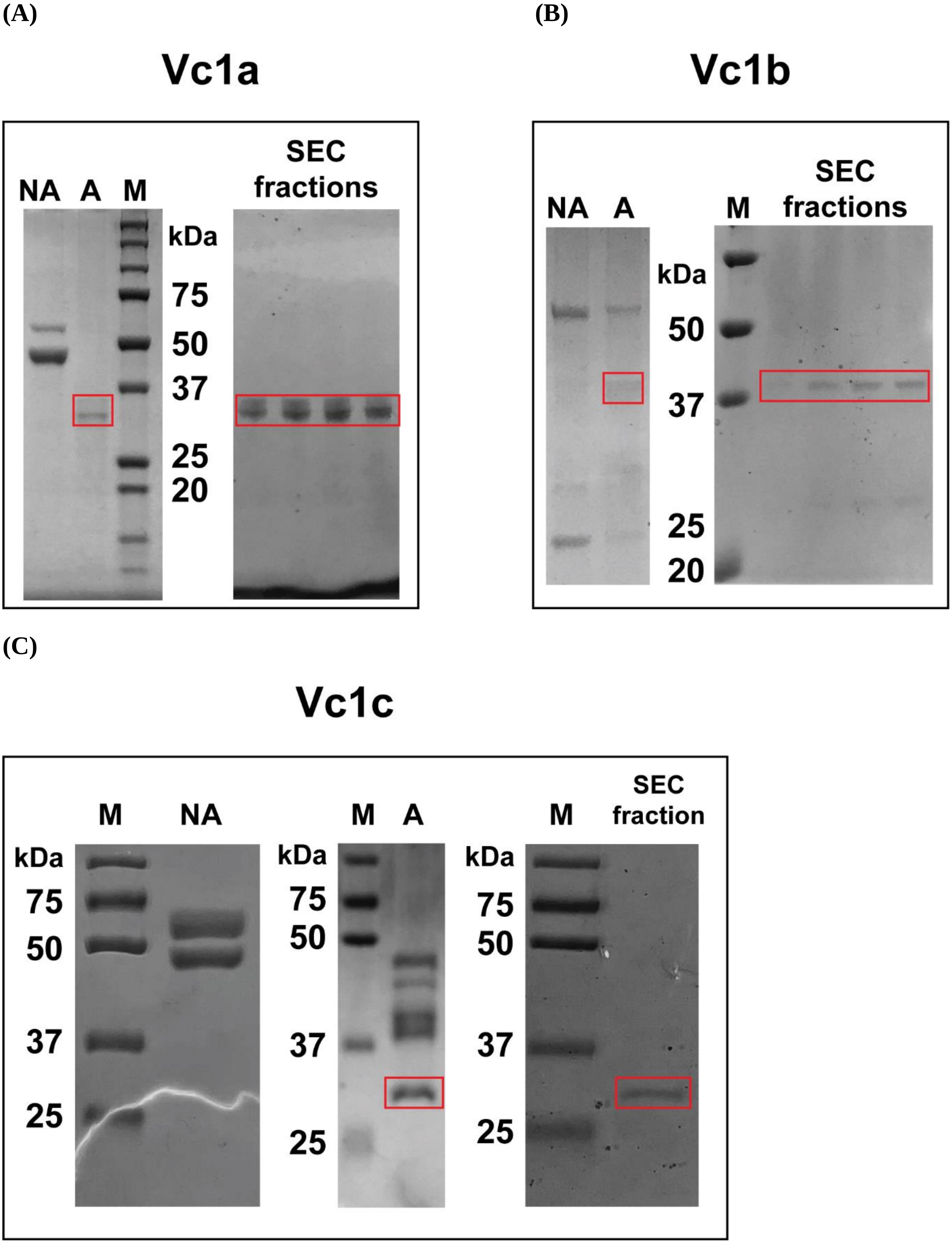

Figure S9. SDS-PAGE analysis of purification and activation of Vc1a (A), Vc1b (B), and Vc1c (C).

The purified activated forms of each enzyme are framed in red boxes. NA: no activation, A: activated, M: protein ladder/marker.

Table S1. RMSD of AEP proenzyme.

Enzyme	Human legumain	Rat legumain	Hamster legumain	OaAEP1b	AtLEGγ	VcAEP	Butelase1	VyPAL2	Butelase2	Butelase2-G252V	Butelase2-G252V/P183A
**Human legumain**	-										
**Rat legumain**	0.753	-									
**Hamster legumain**	0.578	0.602	-								
**OaAEP1b**	0.878	0.694	0.884	-							
**AtLEGγ**	0.962	0.791	1.086	0.642	-						
**VcAEP**	1.077	0.762	0.978	0.549	0.572	-					
**Butelase1**	0.85	0.706	0.82	0.517	0.66	0.597	-				
**VyPAL2**	0.827	0.85	0.769	1.182	0.828	0.812	0.714	-			
**Butelase2**	1.091	0.783	0.812	0.654	0.497	0.546	0.718	0.87	-		
**Butelase2-G252V**	1.023	0.769	0.811	0.684	0.574	0.524	0.752	0.831	0.195	-	
**Butelase2-G252V/P183A**	1.021	0.745	0.84	0.647	0.556	0.596	0.724	0.854	0.269	0.306	-

Table S2. RMSD of active form.

Enzyme	Hamster legumain	Human legumain	HaAEP	AtLEGγ
**Hamster legumain**	-			
**Human legumain**	0.204	-		
**HaAEP**	0.598	0.569	-	
**AtLEGγ**	0.636	0.601	0.297	-
